# Mechanisms of inflammatory response in secondary brain injury: a review of computational approaches, challenges, and future directions

**DOI:** 10.1007/s10237-026-02074-6

**Published:** 2026-06-03

**Authors:** Fatemeh Jafarabadi, Meenal Datta, Maria A. Holland

**Affiliations:** 1 Bioengineering Graduate Program, University of Notre Dame, Notre Dame, 46556 USA; 2 Department of Aerospace and Mechanical Engineering, University of Notre Dame, Notre Dame, 46556 USA

**Keywords:** Impact, Inflammation, Deformation, Edema, Immune cells, Numerical models

## Abstract

Traumatic brain injury (TBI) and the resulting brain damage and dysfunction are one of the leading causes of death for individuals under 40 worldwide. TBI can occur due to any external force causing deformation in the brain, and it involves a complicated timeline of the initial mechanical damage followed by a subsequent inflammatory response. The details and extent of the effects of TBI on the brain are understudied; however, researchers have developed a vast array of experimental and computational models in order to investigate the complex physiological dynamics and interactions in TBI. This review summarizes the hallmarks of TBI, focusing on the inflammatory response mechanisms within the secondary injury and how they have been modeled computationally. Finally, we highlight potential areas of improvement for computational models of TBI to enhance their relevance for translational and clinical research.

## Background

Traumatic brain injuries (TBI) resulting from car accidents, sports activities, and falls are a significant health issue, affecting approximately 7 million people each year and leading to a substantial number of injury-related deaths, long-term disabilities, and the development of neurodegenerative diseases such as Parkinson’s Disease, Alzheimer’s Disease, Chronic Traumatic Encephalopathy, and dementia (Kimelberg 1995; Ayata and Ropper 2002; Bramlett and Dietrich 2015; Maas et al. 2017). In addition, TBI can significantly increase the likelihood of developing psychiatric disorders; even mild TBI can double or triple the risk of depression and anxiety disorders, such as post-traumatic stress disorder, which can further impact a patient’s quality of life (Haarbauer-Krupa et al. 2021; Ghaith et al. 2022).

**Fig. 1:**
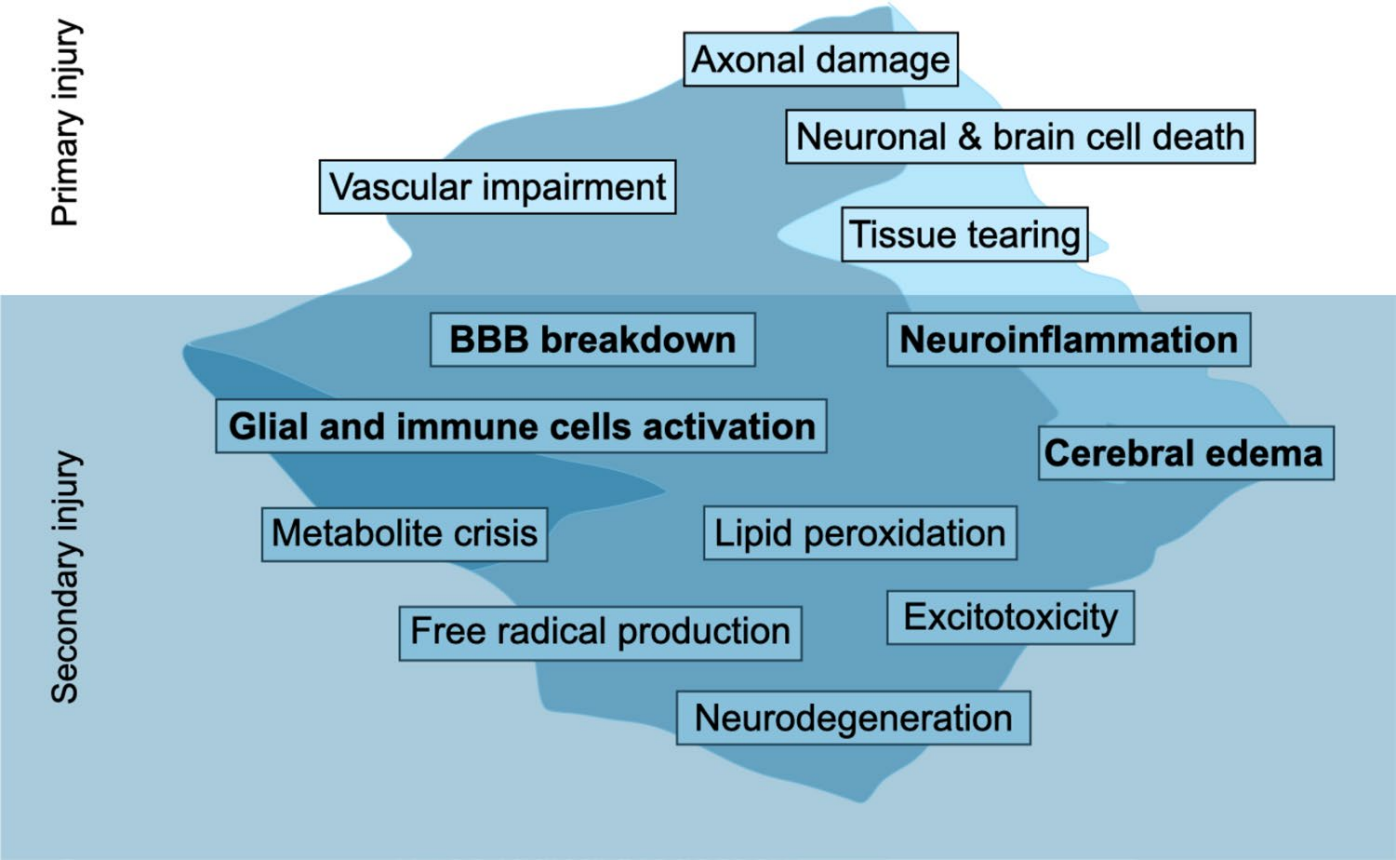
Visible primary injury versus the overlooked secondary injury in the ‘iceberg’ of TBI. While primary injury mechanisms (above waterline) are readily apparent, the more severe secondary injury cascade (below waterline) drive long-term neurological deterioration (Jarrahi et al. 2020; Nwafor et al. 2022; De Macedo Filho et al. 2024). In this paper we will focus on some of these secondary injury processes (shown in bold).

TBI is a multifaceted disease process, encompassing both immediate and delayed pathophysiological events (Kimelberg 1995; Bareyre et al. 1997; Ayata and Ropper 2002; Unterberg et al. 2004; Meaney et al. 2014; Afzal and Zaidi 2014; Vos and Diaz-Arrastia 2015; Ang 2016; Ghaith et al. 2022). The injury results from not only the initial mechanical impact (primary injury) but also the subsequent complex multi-component secondary injury (e.g. inflammatory processes). Early and successful TBI management requires understanding the secondary injury processes and the following immune activity (Fig. 1).

The primary injury occurs when the head is hit by a stationary or moving object, and the resulting impulsive load is transmitted to the brain tissue, causing damage (Sayed et al. 2008; Afzal and Zaidi 2014). In these dynamic impacts, the type of acceleration that causes injury is either translational, rotational, or oscillatory (Cinelli et al. 2019). This motion can cause damage to the blood vessels, axons, neurons, and glial cells in a focal, multifocal, or diffuse pattern of involvement (Sayed et al. 2008; Pawitan 2022; Ghaith et al. 2022). The mechanical response of the brain to this motion depends on its material properties and biological structure, which vary within the brain, between individuals, and throughout life.

Inflammatory response as a part of the secondary injury refers to cellular and molecular events that occur hours, days, and even years after the primary injury and can extend the damage by inducing aberrant and reactionary cellular and molecular activity. In addition to cell death, these mechanisms lead to subsequent tissue damage, blood vessel disruption, diffuse neuronal or axonal injury, plasticity, and atrophy, contributing to the morbidity that follows the trauma (Gentleman et al. 2004; Harting et al. 2008; Cloots et al. 2013; Jayakumar et al. 2014; Balu 2014; Prabhakar et al. 2014). While the primary injury is defined by the passive response of brain tissue, on the longer time scales of inflammatory response, cellular mechanotransduction mechanisms can also impact mechanobiological responses to external mechanical forces (Sayed et al. 2008; Meaney et al. 2014; Keating and Cullen 2021).

Clinical symptoms of TBI include dizziness, headache, loss of consciousness, and deficits in attention, memory, and motor skills. Even if these symptoms improve with time, the microscale damage may persist and increase the likelihood of future neurodegenerative disease (Ayata and Ropper 2002; Bramlett and Dietrich 2015). Both primary and secondary injury components can cause symptoms to varying extents depending on several factors, including the severity of the injury, the impact location, individual patient characteristics, and immune response.

Currently, recognized prognostic factors, such as initial injury severity, lesion volume, and patient age, explain only a portion of the variability in recovery outcomes among individuals with TBI, such as cognitive, physical, and psychological functions, suggesting that secondary injury processes likely contribute to this variation (Bramlett and Dietrich 2015; Munter et al. 2021; Déry et al. 2023). As a result, a better understanding of the relationship between mechanical loading and subsequent damage due to inflammation is necessary to improve the prevention, diagnosis, monitoring, and treatment of TBI.

To achieve this, computational modeling has become a critical tool for exploring the intricate physiological dynamics of TBI (Bayly et al. 2005; Chatelin et al. 2011; Agoston 2017; Amponsah et al. 2024). Computational models are extensively employed in research to quantify brain tissue deformation and injury severity. Furthermore, they provide a foundation for potential applications, such as the design of helmets to enhance safety (Post et al. 2013; Meehan et al. 2022). However, there are some limitations in accurately capturing the full biological complexity of TBI; these models often simplify the interactions between biomechanics, inflammation, and cellular responses, which are critical for understanding long-term outcomes (Cloots et al. 2008; Taib et al. 2017; Mohamed et al. 2020).

In this review, we focus on the mechanisms underlying the damage caused by inflammatory responses in TBI and discuss how these processes can be integrated into computational models to represent the complex, multiphase (solid and fluid), and multiscale nature of TBI. Capturing these intricate processes is essential for advancing our understanding of TBI pathophysiology, developing targeted prevention and mitigation measures, improving therapeutic strategies, and refining predictive tools for recovery outcomes. Current computational approaches, however, often fall short because they lack integration of key factors, including multiscale coupling from molecular (e.g., inflammatory mediators) to cellular (e.g., blood-brain-barrier permeability and glial cell activation) and tissue levels; patient-specific modeling that accounts for mechanical heterogeneity; combined nonlinear constitutive material models (poroelastic and viscoelastic); and repetitive mild injuries. We also highlight the potential of computational frameworks, incorporating insights from experimental investigations and clinical observations, in bridging the gap in understanding between primary injury and the resulting secondary inflammatory response in TBI.

### Biomechanics of TBI

Most brain injuries typically occur not due to direct impact on the brain itself, but rather as a result of forces transmitted through the skull (Sayed et al. 2008). Depending on the incident, different types of internal forces can occur (Keating and Cullen 2021). For example, a shock wave from an explosion can immediately alter the intracranial pressure inside the skull (Smith 2008; Kawoos et al. 2015). Additionally, the sudden movement of the head can cause acceleration or deceleration, resulting in compression, stretching, and shear forces at various points within the brain or surrounding structures (Shuck and Advani 1972; Laplaca et al. 2005; Keating and Cullen 2021).

The types of injuries seen in the brain can be either focal or diffuse (Vos and Diaz-Arrastia 2015). Focal brain injuries are typically severe and starkly visible both during autopsies and via non-invasive imaging. They are mainly caused by direct blows to the head that produce localized damage through compression, laceration, or contusion of brain tissue at either the site of impact (coup), on the opposite side (contrecoup), or both (Andriessen et al. 2010; Rauchman et al. 2022; Freire et al. 2023). Additionally, focal injuries could arise from cavitation effects. This mechanism involves rapid pressure fluctuations that cause the formation and collapse of micro-cavities within the cerebrospinal fluid or brain tissue, producing focal damage even in the absence of direct contact forces (Salzar et al. 2017; Finan et al. 2024). The extent of tissue damage is directly proportional to the peak pressure achieved during the injury period. Diffuse injuries are often associated with rotational head movement, which is dangerous because brain tissue is more susceptible to the shearing forces than tensile or compressive forces (Shuck and Advani 1972; Bandak 1997; Laplaca et al. 2005; Ganpule et al. 2013; Gupta et al. 2017).

### Computational head models

Computational methods have been used extensively to simulate TBI for many years. The aim is to accurately reproduce the impact and predict the resulting brain damage, which would assist in diagnosing injuries and early detection of long-term damage to implement early treatments and enhance preventative measures (Panzer et al. 2012; Gupta et al. 2017; Madhukar and Ostoja-Starzewski 2019; Bayly et al. 2021a; Dagro et al. 2021; Hajiaghamemar and Margulies 2021; Pawitan 2022).

Numerical models for predicting head injury date back to the 1970s and early 1980s (Ward and Thompson 1975; Nahum et al. 1977). However, the most widely used and anatomically detailed finite element (FE) head models have been developed and refined primarily in the 2010s and 2020s (Neal and Kerckhoffs 2010; Yang et al. 2014; Hajiaghamemar and Margulies 2021). This progress has been driven by advances in computational power, high-resolution imaging, and the availability of new *in vivo* and *in situ* experimental datasets for model validation and material property characterization (Zhao et al. 2018; Bayly et al. 2021b; Lyu et al. 2022; Rycman et al. 2024). Modern FE head models now incorporate intricate anatomical features such as sulci, gyri, and regional differentiation between white and gray matter (Horgan and Gilchrist 2003; Sullivan et al. 2015; Li et al. 2021). These models are used to investigate mechanisms of primary brain damage and predict its potential outcomes, such as skull fracture, axonal injury, and brain tissue damage, to inform the design of protective equipment, such as helmets and automotive safety systems.

The ongoing refinement of geometric features and material models continues to enhance the fidelity of TBI simulations (Yang et al. 2014; Sullivan et al. 2015; Wheatley et al. 2015; Li et al. 2021; Amponsah et al. 2024). This includes improvements in model validation techniques (Jiang et al. 2023), skull-brain interface modeling (Kailash et al. 2023; Goodman et al. 2024), and injury prediction parameters (Wu et al. 2022). These advancements are critical in addressing vital clinical questions and providing relevant patient-specific predictions. As the field progresses, researchers face the challenge of balancing increased anatomical detail and accuracy with computational efficiency, in order to create models that are both highly representative of real-world head injuries and practical for widespread use in research and clinical applications. However, despite these advancements, most FE head models largely emphasize mechanical predictors of tissue loading in primary damage. They are not designed to describe the progression of the secondary injury cascade, including inflammatory response. This highlights the potential of mechanistic computational models that integrate biomechanical loading with specific pathological mechanisms to define functional dependencies and support a more integrated understanding of traumatic brain injury progression.

## Mechanisms of inflammatory response

**Fig. 2:**
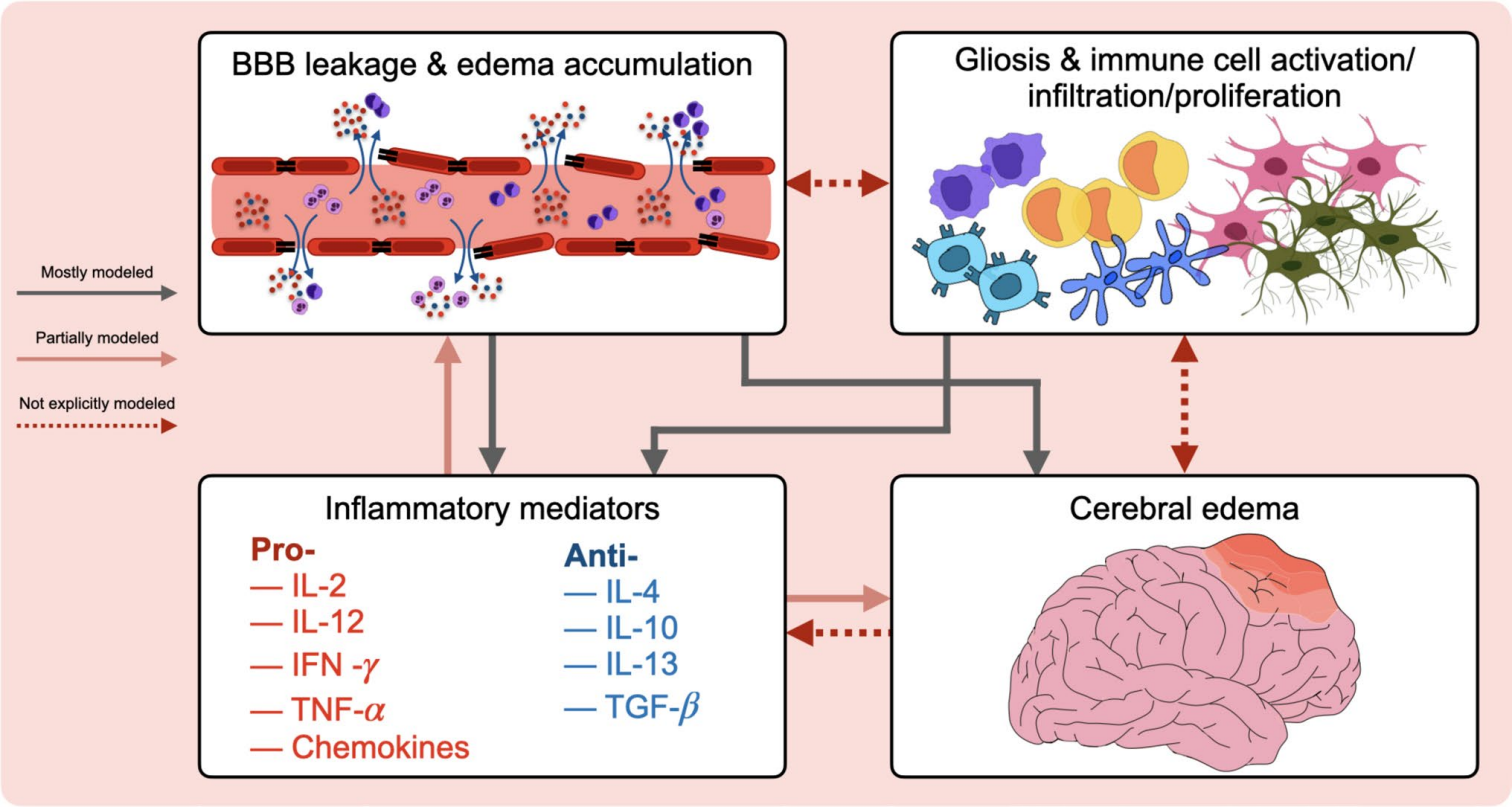
Molecular and cellular activity following TBI. Blood-brain barrier leakage triggers a complex inflammatory response characterized by the release of pro- and anti-inflammatory cytokines; glial cell activation and immune cell activation, infiltration, and proliferation; and cerebral edema. Solid gray arrows indicate interactions that have been explicitly represented in existing computational models, solid red arrows denote interactions that are only partially or indirectly modeled, and dotted red arrows highlight biologically established couplings that remain understudied in current modeling frameworks.

TBI results in the disruption of the blood-brain barrier (BBB) and triggers a complex cascade of inflammatory reactions involving cytokine release, immune cell infiltration and proliferation, and glial cell activation. These processes contribute to edema accumulation and the progression of damage (Fig. 2).

### Blood-brain barrier leakage

The BBB is a complicated structure that regulates the exchange of all substances between blood plasma and the brain interstitial fluid, permitting only the passage of small lipophilic molecules while excluding most others. By forming a selective physical barrier, it regulates the cerebrospinal fluid (CSF) balance and the central nervous system (CNS) microenvironment, maintaining homeostasis (Shlosberg et al. 2010; Alam et al. 2020; Profaci et al. 2020). The BBB is composed of several cell types, including endothelial cells, astrocytes, microglia, and pericytes (Correale and Villa 2009). Historically, the BBB was considered primarily a passive barrier separating the CNS from the peripheral immune system and preventing the entry of infectious agents and immune cells (Muldoon et al. 2013; Ampie and McGavern 2022). However, new observations indicate that the BBB is not passive from an immunological point of view; rather it also plays an active role in the immune response of the CNS after injuries (Cash and Theus 2020; Raslan et al. 2010). Trauma to the brain disrupts the BBB, permitting the leakage of brain proteins and their breakdown products into the circulation. In some cases, antibodies are generated and circulated in the blood for an extended period of time, presenting a new class of biomarkers (Smith et al. 2013; Shahim et al. 2018). The disruption also assists in the recruitment of circulating neutrophils, macrophages, and lymphocytes to the injured site (Chodobski et al. 2011; Cash and Theus 2020).

The blood-brain barrier’s response to TBI has been extensively studied in animal models, particularly following focal impact injuries (Raslan et al. 2010; Tchantchou and Zhang 2013; Thal and Neuhaus 2014). These studies suggest that TBIs are associated with an upregulation of vascular endothelial growth factor (VEGF) and matrix metalloproteinase-9 (MMP-9), resulting in BBB disruption and nerve damage (Nag et al. 2002; Dore-Duffy et al. 2007). This disruption is bi-phasic, with an initial peak in vascular permeability, within hours of injury, followed by a second phase of increased permeability after 3-7 days (Obermeier et al. 2016; Laskowitz and Grant 2016). While clinical data indicate that BBB permeability typically normalizes within days or weeks, some diffuse injuries show persistent disruption months or even years later (Stahel et al. 2001; Tomkins et al. 2011; Keating and Cullen 2021). However, it remains unclear whether prolonged BBB dysfunction stems from direct mechanical damage, secondary pathological cascades such as inflammation and oxidative stress, or both.

#### Current computational approaches and gaps in understanding

Computational modeling offers a promising avenue to address these uncertainties by allowing controlled investigations of BBB pathology across multiple scales. Currently, computational models of the BBB cover a wide range of scales, from molecular dynamics and docking that resolve transporter behavior to mechanistic continuum-based FE models at the tissue and organ level (Garg and Verma 2006; Martins et al. 2012; Heye et al. 2016; Huang et al. 2024; Alves et al. 2025). However, their application to the highly dynamic and heterogeneous BBB disruption following TBI remains limited and often fails to capture the spatiotemporal evolution of tight junction degradation and the distinct phases of cerebral edema formation. This wide range of scales is valuable but exposes recurring shortcomings. High-resolution molecular-level models provide fine structural detail but are computationally constrained, limiting models to small scales and short durations and often simplifying the complex lipid environment of the cell membrane by representing it with a single lipid type or a uniform component (Carpenter et al. 2014). Furthermore, these fine-scale models fail to explicitly incorporate TBI-specific mechanistic couplings, such as the direct, transient influence of impact-induced shear stress and rapid tissue stretch on the endothelial cell membrane remodeling, which drives the activation of mechanosensitive channels and the temporal progression of permeability in injury (Cucullo et al. 2011; Partyka et al. 2017; Kim and Kwon 2018; Hansen et al. 2024). Conversely, larger-scale approaches suffer from a critical lack of underlying molecular detail, overlooking essential dynamics like the interactions between activated microglia, infiltrated immune cells, and the neuroinflammation feedback loop, along with the influence of dynamic biochemical factors. This is particularly critical in TBI, where the rapid, spatially heterogeneous mechanical breakage of the BBB must be coupled to the slower, sustained immune response it subsequently triggers. Many models also depend on poorly constrained parameters (anisotropy, lipid composition, transporter kinetics, and immune signaling rates), producing non-identifiable parameter sets and reducing the predictive power (Shityakov and Förster 2018).

To address these limitations specific to TBI’s dynamic and mechanically-driven nature, and to manage the extreme computational and parameter uncertainty of these coupled systems, future efforts should adopt a hierarchical, hybrid framework that explicitly links the different scales. This hybrid approach, however, necessitates careful simplification, focusing only on the dominant factors of BBB dynamics, such as mechanically-induced tight junction deformation and cytokine signaling, to ensure the resulting multi-physics model remains computationally tractable and its complex parameters are biologically constrained. Practical coupling strategies include one-way up-scaling methods, such as homogenization or parameter passing, which can translate information from molecular dynamics of tight junction proteins to tissue-level BBB permeability in TBI. Another key strategy involves computational surrogates for expensive fine-scale simulations, encompassing techniques like reduced-order models and physics-informed neural networks. Finally, a more advanced approach is two-way coupling where macroscale shear or strain alters mesoscale kinetics (Garg and Verma 2006; Huang et al. 2024). Additionally, a rigorous validation pipeline and standardized benchmark datasets will be necessary to quantify predictive uncertainty and guide translational use.

While the hierarchical strategies described above currently serve as conceptual recommendations, there are potential technical challenges for their implementation, including the vast time-scale disparity involved in mapping the millisecond molecular dynamics of junctional proteins to the days- and weeks-long progression of clinical edema.

### Inflammatory mediators

Following cellular injury, various biomarkers such as S100B, neuron-specific enolase (NSE), glial fibrillary acidic protein (GFAP), and ubiquitin C-terminal hydrolase L1 (UCH-L1) are released, where they serve as indicators of neuronal and glial activation and damage in clinical assessments of brain injury (Hossain et al. 2024). The concentration of these markers peaks within a few hours of injury and then gradually declines, depending on the specific half-life of the biomarker in the blood (Diaz-Arrastia et al. 2014). Neuroinflammation and the emergence of cytokines are relatively slower processes than the early burst of clinical biomarkers, occurring during the later stages of secondary injury (Helmy et al. 2011).

Neuroinflammation is a critical component of the secondary injury cascade after TBI. The inflammatory response in the CNS begins shortly after an injury, with a significant increase in pro-inflammatory mediators at the injury site, while anti-inflammatory cytokines remain unchanged (Adrian et al. 2016). The pro-inflammatory cytokines include interleukins IL-1$\alpha$, IL-1$\beta$, and IL-6, tumor necrosis factor-$\alpha$ (TNF-$\alpha$), and interferon-$\gamma$, which have been linked to poor functional and neurological outcomes (Ozen et al. 2020). These cytokines serve as master initiators of the inflammatory response by upregulating adhesion molecules, releasing chemotactic chemokines, and activating cells of the adaptive immune system (Helmy et al. 2011). Conversely, anti-inflammatory cytokines, such as IL-4, IL-10, IL-13, and transforming growth factor-$\beta$ (TGF-$\beta$), decrease inflammatory and cytotoxic reactions during the resolution phase, which varies between days and weeks (Cederberg and Siesjö 2010; Woodcock and Morganti-Kossmann 2013; Adrian et al. 2016; Needham et al. 2019). High levels of cytokines have been measured in both CSF, peaking within the first days after injury, and in the blood serum, peaking a few days post-injury (Ransohoff and Brown 2012; Lescot et al. 2010). After the initial peaks, elevated levels of several cytokines were measured in the serum for more than three months, indicating chronic inflammation post-TBI. The severity of TBI affects the peak and duration of cytokine secretion (Helmy et al. 2011; Adrian et al. 2016).

Molecular measurements of blood and CSF, which reveal the time course of biomarkers of TBI, typically start upon the patient’s admission to the hospital and continue at various intervals for a few days or a week (Zetterberg et al. 2013; Shahim et al. 2018). Thus, the first measures in the sequence represent a time point of at least a few hours after injury; more data on the very early kinetics of biomarkers in human subjects need to be collected. Researchers typically use a combination of molecular, histological, and biochemical techniques to quantify cytokines, chemokines, and cellular markers associated with neuroinflammation (Gentleman et al. 2004; Woodcock and Morganti-Kossmann 2013; Wangler et al. 2022; Zhang et al. 2023b). Animal models enable researchers to examine the temporal profile and release of inflammatory mediators following TBIs. One study found an early increase in IL-1$\beta$, while the expression of both pro- and anti-inflammatory mediators was resolved within a week (Taib et al. 2017). In another study, researchers exposed neuronal cultures derived from human stem cells to IL-6 and TNF-$\alpha$ to simulate post-TBI conditions, finding that these cytokines elicited a significant and measurable increase in cytokine and chemokine production in a dose- and time-dependent manner (Thelin et al. 2018).

#### Current computational approaches & gaps in understanding

Ordinary differential equation (ODE) models have proven useful for capturing the temporal interactions among pro- and anti-inflammatory cytokines and tissue damage in TBI, enabling predictions of population-average cytokine trajectories and *in silico* evaluation of therapeutic perturbations (Vaughan et al. 2018; Constantine et al. 2016). However, ODEs fundamentally lack spatial resolution. Inflammation is not a uniform process; it unfolds within localized microenvironments where microglial activation, astrocytosis (reactive proliferation and hypertrophy), and edema accumulation occur. On the other hand, data-driven dimension-reduction strategies can refine measurements of numerous biological mediators into a smaller number of prognostic signatures that are most strongly correlated with the injury (Kumar et al. 2016b). These reduced dimensions can help prioritize targets and constrain parameter spaces for mechanistic models; however, they combine multiple biological signals and thus lack direct mechanistic interpretation, and their results are sensitive to preprocessing, assay variability, and cohort size, which can reduce generalization.

A hybrid computational workflow is therefore necessary for future models to effectively handle this complexity and ensure clinical relevance. This approach should first apply dimensionality reduction to condense high-dimensional measurements of inflammatory mediators, derived from *in vivo* and clinical samples, into a manageable set of key variables for the model (Kumar et al. 2016b; Chiu et al. 2016; Prabhakar et al. 2022). Implementing these data-driven variables within spatially resolved partial differential equations (PDEs), such as reaction-diffusion and reaction-advection models, across 2D and 3D frameworks, allows us to both simplify the complexity of the biological system and simultaneously strengthen model validation using relevant experimental and clinical evidence. Additionally, this integration provides the necessary spatial context missing in ODEs while justifying the data-driven simplification within a mechanistic framework.

### Glial cell activation & immune cell infiltration/proliferation

Following TBI, the cytokine response comes from both immune cells as well as a diverse set of other immunocompetent cell types such as astrocytes, microglia, endothelial cells, and even neurons (Donat et al. 2017). After the initial mechanical trauma causes direct damage, the microglia and astrocytes initiate a cascade of immune events directed by releasing damage-associated molecular patterns (DAMPs), stimulating nonspecific immune responses and resulting in the recruitment of peripheral neutrophils and monocyte-derived macrophages into the brain from the blood (Karve et al. 2016; Alam et al. 2020). Depending on the nature of the impact, various factors such as traumatic contusion, diffuse injury, or raised intracranial pressure can contribute to the resulting inflammatory response with potentially distinct cellular patterns (Adrian et al. 2016).

Post-TBI, astrocytes become reactive and undergo a phenotypic change, which results in astrocyte swelling (Jayakumar et al. 2014; Shanaki-Bavarsad et al. 2022). Astrocytes rapidly produce anti-inflammatory molecules in the injured brain, which could be one of the mechanisms for reversing microglial activation in TBI. This highlights the complexity of the astrocyte response, as they both initiate and respond to inflammation. Furthermore, astrocytes support neuronal survival by providing growth factors and nutrients to neurons and maintaining extracellular fluid homeostasis (Burda et al. 2016; Constantakis et al. 2023).

While astrocytes perform critical and beneficial functions following brain injury, the reactive changes they undergo, including swelling, can also contribute to disease pathology, leaving their overall impact on recovery and progression of TBI uncertain (Adrian et al. 2016). Most studies on diffuse brain injury have focused on axonal damage in neurons, but astrocytes also exhibit significant responses to mechanical stress, including sub-cellular signaling pathways that may drive cell damage and death even without visible axonal injury (Laplaca and Thibault 1997; Laplaca et al. 2005; Lu et al. 2022; Constantakis et al. 2023). Traditionally considered less vulnerable than neurons, astrocytes can amplify damage through several mechanisms: transmitting mechanical forces to neurons, propagating Ca$^{2+}$ waves, and contributing to excitotoxicity via altered neurotransmitters and extracellular ion concentrations (Needham et al. 2019; Muñoz-Ballester and Robel 2023).

Microglia are phagocytic cells that serve as the primary resident immune cells in the CNS (Karve et al. 2016). After TBI, microglia provide crucial immune surveillance and can initiate brain-specific inflammatory responses by changing their morphology and expressing pattern recognition receptors (PRRs) (Colonna and Butovsky 2017; Donat et al. 2017). Activation of these PRRs induces cytokine release in the brain, and these cytokines induce multiple downstream effects, including changes in cerebral blood flow, increases in BBB permeability, and an influx of peripheral cells into the brain (Sayed et al. 2008; Adrian et al. 2016). In mild injuries, a mixed population of microglial phenotypes with pro-inflammatory cytokines and chemokines is increased from 6 hours to 3 days post-injury, recruiting immune cells into the CNS and promoting astrocytosis (Unterberg et al. 2004; Karve et al. 2016).

While glial cells, including both astrocytes and microglia, play a central role in neuroinflammation, neutrophils and macrophages are also of interest as key early responders that shape the injury environment. Additionally, the meninges contain a variety of immune cells, originating from the skull and vertebral bone marrow, that in the case of an injury infiltrate the CNS. While the skull bone marrow-derived immune cells are not as abundant as the peripheral bone marrow-derived cells, they localize to the edge of the trauma site and predominantly express anti-inflammatory phenotypes in contrast to the peripheral cells that accumulate closer to the core of the trauma site and often exhibit a pro-inflammatory role (Cash and Theus 2020; Cugurra et al. 2021).

The involvement of neutrophils differs between focal and diffuse injuries. In experimental mouse models with focal injury, the infiltration of neutrophils appears early, peaking within a few days, followed by the migration of other cell types to the site of injury (Balu 2014; Von Leden et al. 2019). However, pre-clinical studies of diffuse injury in mice indicated little to no neutrophil infiltration, with early cellular responses characterized by prominent microglial accumulation and astrocytosis, particularly evident in the white matter tracts (Herz et al. 2015; Alam et al. 2020). Furthermore, studies using neutrophil-depleted mice have suggested that neutrophils are involved in cerebral edema and contribute to cell death and tissue loss following diffuse TBI in mice. Thus, early neutrophil depletion effectively reduces the tissue loss in the secondary injury (Kenne et al. 2012; Liu et al. 2018).

Finally, TBI triggers a complex and heterogeneous macrophage response over time that localizes near the injury site (Yu et al. 2022). Initially, it was thought that macrophages expressing both pro-inflammatory (M1-like) and anti-inflammatory (M2-like) phenotypic markers (Hsieh et al. 2013; Turtzo et al. 2014) congregate near the injury, with the M2-like macrophages diminishing and leading to a predominance of M1-like cells, which are associated with increased neurodegeneration (Kumar et al. 2016a). However, single-cell analyses showed that macrophages responding to acute TBI do not segregate into distinct inflammatory or reparative subsets but instead exhibit a mix of inflammatory (M1-like) and reparative (M2-like) markers within the same cell, displaying characteristics of both monocytes and embryonic microglia following secondary injury (Kim et al. 2016; Barrett et al. 2017; Specht et al. 2021; Feng et al. 2021). These findings highlight the complexity of macrophage dynamics in TBI and underscore the need to consider their nuanced roles when developing therapeutic strategies.

Advanced imaging techniques, including high-resolution microscopy and *in vivo* imaging, have enabled real-time visualization of microglial activation, migration, and cellular interactions, revealing crucial temporal aspects of microglia responses, such as the transition between pro- and anti-inflammatory states and their role in tissue repair (Grovola et al. 2023; Larrea et al. 2023). Various experimental studies, primarily using mouse models, have investigated the role of glial cells and innate immune cells in the inflammatory response after TBI (Harting et al. 2008; Donat et al. 2017). These models have provided valuable insights into cell-type-specific transcriptional responses and the dynamic nature of glial activation post-TBI (Todd et al. 2021; Bray et al. 2022; Wangler et al. 2022). However, preclinical modeling of this response remains challenging due to the heterogeneity of glial states, species-specific differences, and the dynamic interplay between injury severity and inflammatory cascades (Zhang et al. 2023b).

#### Current computational approaches & gaps in understanding

While computational neuroscience has traditionally focused on neuronal function, ODE-based models have been instrumental in formalizing the temporal feedback dynamics of glial-mediated neuroinflammation following TBI (Postnov et al. 2007; Silchenko and Tass 2013; Anderson et al. 2015). These existing frameworks capture the time courses of bulk cytokine-glial interactions, providing insights into glial cell polarization and the cytokine feedback loop that drives inflammation based on both controlled *in vivo* experiments and clinical data from the first five days post-injury (Anderson et al. 2015; Vaughan et al. 2018). However, methodological limits hinder the translation of these models to clinically relevant scenarios. Current ODE models generally fail to represent critical biological complexities. These include stochastic single-cell variability, mechanotransduction, and the reciprocal coupling between resident glia and infiltrating immune cells. Moreover, ODEs struggle with spatial heterogeneity (Salman and Mohanty 2025), which is better captured by PDE models that have been successfully applied to neuroinflammation in non-TBI conditions. However, even these PDE models are predominantly a set of reaction-diffusion equations which lack the crucial biomechanical coupling required to simulate the multi-physical nature of TBI (Khajanchi and Nieto 2021). Furthermore, both ODE and PDE models are constrained by fundamental computational challenges such as high parameter dimensionality, making calibration and validation difficult with sparse, limited-resolution histological or imaging data available.

To effectively address these gaps, future mathematical frameworks must leverage spatially explicit models (PDEs or hybrid agent-based/continuum models) to capture local activation; focus on modular integration to facilitate multiscale coupling and mechanochemical feedback; and explicitly represent cellular complexity, such as phenotypic switching and gene regulatory networks, using stochastic rules or Boolean approaches (Subramanian and Sahoo 2022; Irastorza-Valera et al. 2024). Such efforts will target specific aspects of glial activation and immune cell infiltration in TBI. Ultimately, achieving clinically translatable models requires improved experimental design that explicitly links high-resolution immunohistochemistry and longitudinal imaging to provide the rich, spatiotemporal validation data necessary for model calibration and statistical approaches to account for patient-specific uncertainties.

### Cerebral edema

**Fig. 3:**
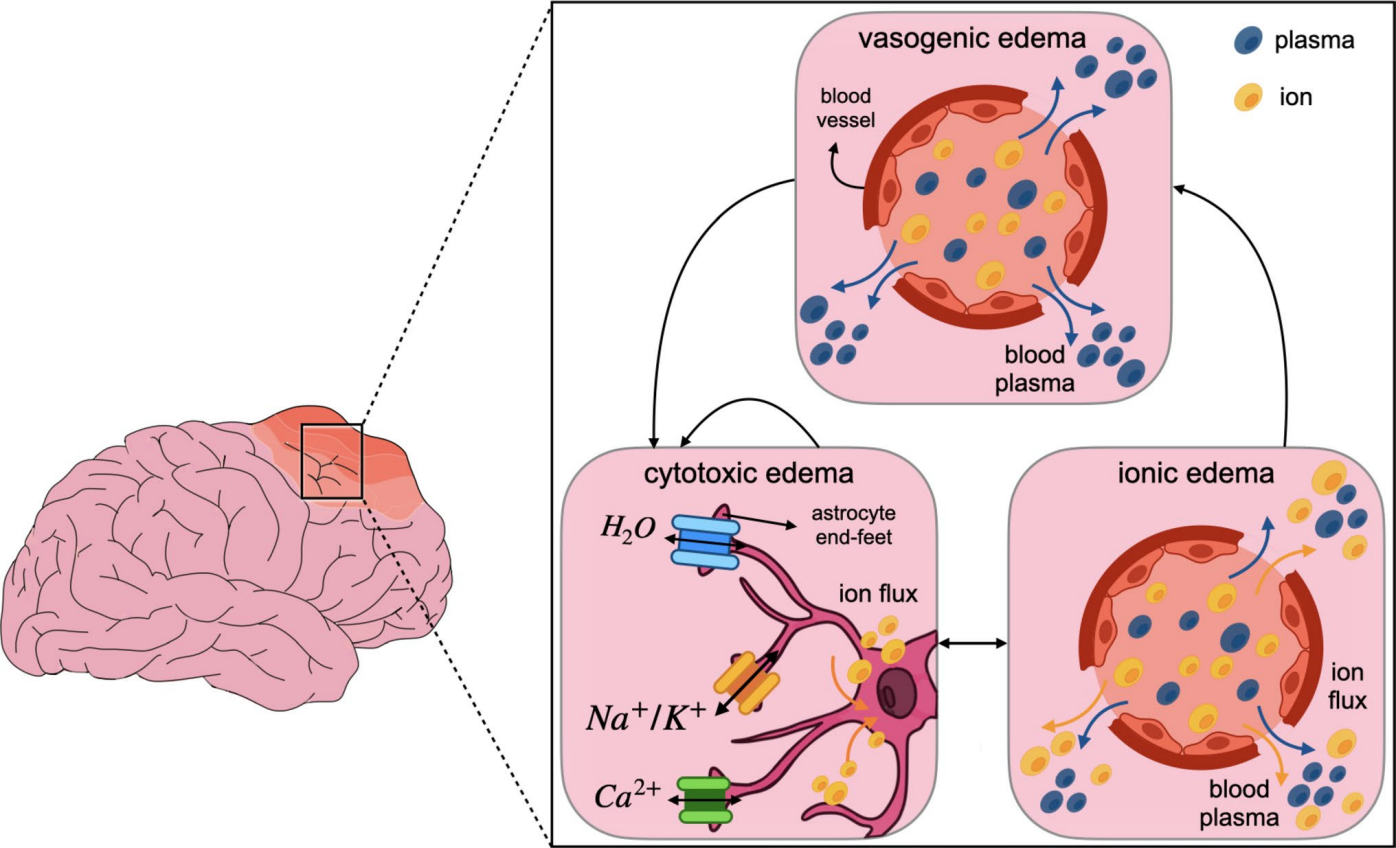
Schematic of three main types of brain edema: vasogenic edema characterized by blood vessel leakage; cytotoxic edema showing astrocyte swelling driven by ion and intracellular water influx; and ionic edema driven by osmotic imbalances and ion shifts across both the vasculature and cell membranes

Cerebral edema, the accumulation of excess fluid within the interstitial spaces of brain tissue, was traditionally categorized into physiological types without molecular or cellular explanations, particularly neglecting the critical interactions at the neurovascular junction, where endothelial cells, astrocytes, neurons, and microglia converge and contribute to inflammation. However, recent developments in our understanding of the molecular mediators of edema formation have led to reevaluating the classical subtypes of cerebral edema. Today, cerebral edema is classified into three main types: vasogenic, cytotoxic, and ionic (Fig. 3) (Kimelberg 1995; Michinaga and Koyama 2015).

Vasogenic edema is characterized by increased BBB permeability due to endothelial dysfunction or inflammatory processes, leading to the extravasation of fluid and proteins into the brain tissue. The abnormal fluid accumulation causes an increase in brain volume because of an enclosed, rigid skull (Simard et al. 2007; Bothwell et al. 2019). Cytotoxic edema, on the other hand, involves cellular swelling due to water accumulation within brain cells, particularly astrocytes (Jayakumar et al. 2014; Burda et al. 2016). This process is mediated by aquaporins, water-specific channels, rather than the ion channels that contribute to other types of edema (Czyżewski et al. 2024). The glymphatic system, a lymphatic-like waste clearance mechanism in the CSN, is facilitated by aquaporin-4 (AQP4) channels and relies on the interplay between CSF and interstitial fluid (ISF). In the brain after TBI, the redistribution of AQP4 on astrocytes results in dysfunction of the glymphatic system, impairment of the excess fluid clearance, and ultimately the formation of edema (Sullan et al. 2018; Ferrara et al. 2022; Peters and Lyketsos 2023; Siri et al. 2024). Cytotoxic edema alone does not cause cerebral edema since it simply involves a shift of osmolytes and water from extracellular to intracellular compartments. However, if the overall fluid balance within the brain is disrupted, cytotoxic edema can contribute to increased brain volume (Michinaga and Koyama 2015). Finally, ionic edema results from a disruption in ion balance, particularly involving sodium and potassium, which triggers osmotic shifts, leading to cerebral edema and exacerbating damage (Simard et al. 2007; Liang et al. 2007; Denes et al. 2010).

The interplay between vasogenic, cytotoxic, and ionic edema creates a dynamic, self-perpetuating cycle of cerebral edema (Fig. 3). This cycle begins with sodium and water fluxes from the bloodstream, which leads to a depletion of extracellular sodium and a gradient in favor of the movement of sodium from the vascular to the tissue compartments. This induces upregulation of ion channels in astrocytes and vascular cells that enable sodium and water to follow this new sodium gradient. Transporting these substances across the vessel wall causes ionic and vasogenic edema, which increases brain mass and volume. Notably, newly arrived sodium can be continuously taken up by astrocytes undergoing cytotoxic edema, driving the formation of further cytotoxic and ionic edema (Pasantes-Morales and Vázquez-Juárez 2012; Stokum et al. 2015; Lu et al. 2022; Datta et al. 2023). Additionally, inflammation can significantly fuel cytotoxic edema by releasing cytokines and chemokines that disrupt astrocyte function. Furthermore, inflammation can cause vasogenic edema, which contributes to the BBB breakdown and intracranial pressure (ICP), indirectly exacerbating cytotoxic edema (Michinaga and Koyama 2015).

The role of mechanics in cerebral edema is a critical area in TBI research. The mechanobiological consequences of edema create a dynamic environment that enhances immune cell recruitment and directs them to the site of inflammation by providing mechanical signals, including increased interstitial fluid pressure gradients, altered matrix stiffness, and changes in local tissue confinement (Pathak and Kumar 2012; Seano et al. 2019). However, cerebral edema can adversely affect the surrounding tissue (Padera et al. 2004; Engelhardt and Coisne 2011; Shigeta et al. 2020). The fixed volume within the skull means that as swollen tissue expands, it exerts a mechanical force on adjacent tissue, increasing tissue pressure. This elevated pressure can displace surrounding tissue and, in extreme cases, leads to necrosis and cell death in the compressed tissue (Maas et al. 2008; Datta et al. 2023). Crucially, if pressure levels are severely high, it can mechanically restrict the drainage of cerebral blood by compressing the intracranial veins (Siri et al. 2024). This circulatory obstruction further increases the fluid volume within the rigid cranial compartment, initiating a dangerous, self-sustaining feedback loop known as intracranial hypertension (Rangel-Castillo et al. 2008; De Simone et al. 2010; Kofke et al. 2020).

Experimental models of TBI have employed various techniques to study inflammatory swelling and cerebral edema, revealing multiple mechanisms and potential therapeutic targets. For instance, studies comparing *in vitro* astrocyte cultures from rats with functional NF-$\kappa$B inactivation to those from wild-type mice have demonstrated that NF-$\kappa$B activation is crucial for post-traumatic astrocyte swelling and brain edema (Pasantes-Morales and Vázquez-Juárez 2012; Stokum et al. 2015; Lu et al. 2022), suggesting that targeting this pathway could potentially reduce edema.

#### Current computational approaches & gaps in understanding

Cerebral edema is complex, and differentiating between vasogenic edema (Bothwell et al. 2019), cytotoxic edema accumulation (Jayakumar et al. 2014; Stokum et al. 2015), and ionic edema (Denes et al. 2010) *in vivo* remains a challenge due to overlapping physiological processes. Traditional methods like wet/dry weight comparisons have provided direct measures of brain water content (Adelson et al. 1996; Flierl et al. 2009; Kane et al. 2012), and modern imaging techniques (Andriessen et al. 2010; Albert-Weißzlig;enberger et al. 2012), both primarily show overall cerebral edema accumulation without making the distinction between underlying edema types. This highlights the need for computational models that can integrate these mechanisms and their individual roles to significantly advance our knowledge base regarding cerebral edema. The fundamental computational limitation is the inability to decouple and quantify these individual fluid transport pathways *in silico*. Current models often successfully capture the bulk tissue deformation and the mechanical impact of overall volume expansion, which is crucial for predicting tissue displacement and ICP dynamics (Ju et al. 2020). However, representing the microscopic biophysical drivers of edema requires far more detailed formulations than most existing continuum models can sustain. One priority is to develop multiscale models that go beyond simple continuum mechanics or molecular dynamics, can distinguish between cellular and interstitial mechanisms of cerebral edema, and predict their relative contributions to overall tissue expansion in TBI. Such efforts must balance biological fidelity with computational feasibility; therefore, the goal is to incorporate the dominant mechanisms relevant to edema evolution, such as the mechanical disruption of tight junctions leading to vasogenic leakage, or the acute failure of ion channels driving cytotoxic swelling, rather than attempting comprehensive reconstructions of all post-injury cascades. These models would enable better assessment of anti-edema and anti-inflammation treatments, helping to determine whether to target cellular volume regulation or interstitial fluid management in different phases of post-TBI cerebral edema.

Fluid regulation and tissue displacement models have been successfully applied to other brain conditions such as hydrocephalus, ischemic stroke, and brain tumors (Vardakis et al. 2016; Chen et al. 2022; Zheng et al. 2024). These models, which incorporate brain vasculature and interstitial spaces, leverage coupled fluid-solid mechanics to identify critical regions of interstitial fluid build-up and assess therapeutic intervention. Despite their complexity, sensitivity to small parameter changes, and challenges in translating simulation results to patient outcomes, their application to TBI-induced edema could provide significant insight into its progression and treatment, despite intentionally simplifying many biochemical details. Additionally, recent advances in multiscale and multiphase modeling of other organs, such as the heart, offer useful strategies for TBI modeling, even though the relevant timescales (heartbeats versus TBI injury progression) are significantly different (Reis et al. 2019; Barnafi et al. 2022). For instance, cardiac edema models have demonstrated how to successfully integrate pathogen-immune interactions with tissue mechanics and patient-specific imaging (Reis et al. 2019). Leveraging these approaches could transition TBI edema modeling from purely mechanical simulations to true bio-transport models that can explicitly predict how cellular volume regulation or interstitial fluid management will impact overall brain volume and therapeutic outcomes at different post-TBI injury phases.

## Other challenges

### Multiscale phenomena

**Fig. 4:**
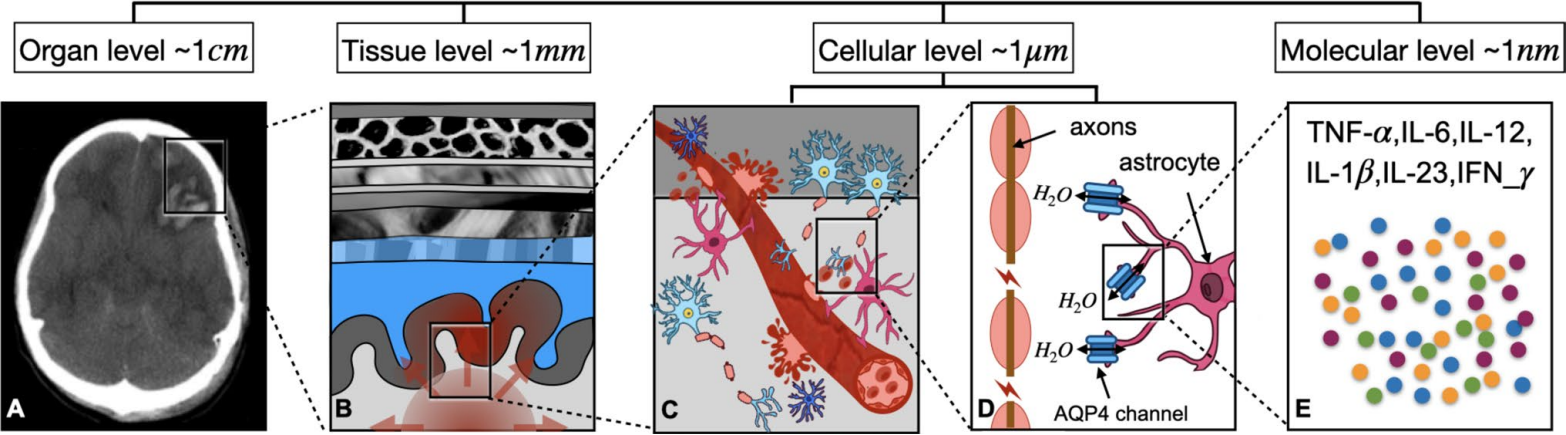
TBI manifests as a multiscale phenomenon, affecting the head and brain across different hierarchical levels (the scan (A) is adapted with permission from Andriessen et al. 2010).

TBI concerns a wide range of length and time scales. At the length scale, it spreads from several centimeters at the organ level, where the mechanical damage is applied, to several nanometers at the molecular level, where the inflammatory processes occur (Fig. 4) (Hemphill et al. 2015; Ghajari et al. 2017; Keating and Cullen 2021). At the temporal scale, immediate mechanical damage and neurological disruption occur within seconds, while the next round of damage from the inflammatory response plays out over the following hours and days, and chronic neurological disorders may even occur years after the initial impact (Ayata and Ropper 2002; Maas et al. 2017; Ghaith et al. 2022). However, most diagnostic models have been developed to study TBI at a single length and time scale; for example, primary head models simulate the rapid ($\sim \hbox {ms}$) macroscopic ($\sim \hbox {cm}$) mechanical deformation of the brain (Madhukar and Ostoja-Starzewski 2019; Giudice et al. 2019). This approach does not capture the mechanisms underlying the progression of damage from tissue to cellular and molecular levels at different time points.

Currently, there is still no well-established relationship between the different length scales in TBI. For instance, finite element head models have been beneficial in demonstrating the link between head movements and tissue deformation in TBI (Cloots et al. 2013; Gupta et al. 2017; Hajiaghamemar and Margulies 2021). But even personalized head models that incorporate mechanical anisotropy use tissue-level injury metrics based on strains transferred to axonal fiber damage rather than directly modeling axonal deformation and intercellular junction mechanics (Montanino et al. 2021). Other effects, such as molecular activity, cell proliferation, and death, should also be explored.

After a mechanical injury to the brain, discrete impairments occur at points where axons deviate from their usual path, often near blood vessels or cell bodies. This suggests that the brain’s microstructural heterogeneity, including factors like microvasculature and axonal orientation, plays a key role in influencing localized stress and strain, thus affecting tissue sensitivity to mechanical loads (Laplaca and Thibault 1997; Laplaca et al. 2005; Cinelli et al. 2019; Montanino et al. 2021; Wu et al. 2022; Zhang et al. 2023a). Multiscale models that account for these structural variations have shown how cellular structures impact localized strain and how these effects propagate over the scales of TBI (Cloots et al. 2013).

Scale is also important when translating from animal models to humans. Primary injury varies significantly in scale across different species due to differences in brain structure and size. For instance, in humans, it can range from millimeters (e.g., small contusions) to centimeters (e.g., large hematomas), while in mice, it occurs on a considerably smaller scale from micrometers to a few millimeters (Awasthi et al. 2024). Secondary injury due to inflammation also exhibits an extensive spatial range, often spreading beyond the immediate site of primary damage, with neuroinflammation, edema, and axonal injury that could affect distant regions of the brain. For instance, in rodent models, the damage could encompass a large portion of the brain hemisphere ($\sim \hbox {mm}$), while in humans, it can involve multiple lobes ($\sim$ tens of cm). Thus, it is crucial to account for the differences in scale across species when extrapolating findings from animals to humans.

Machine Learning (ML) models in TBI research can significantly enhance our understanding of injury mechanisms across multiple scales. ML can bridge the gap between tissue-level deformation predictions derived from FE models, data from imaging modalities (e.g., MRI and microscopy), and cellular or molecular responses, providing a more comprehensive and accurate picture of injury progression (Awasthi et al. 2024). For example, deep learning (DL) models can predict the evolution of localized damage, such as axonal shearing or neuroinflammation, based on the strain and stress distributions at the tissue level (Gassenmaier et al. 2021; Lee et al. 2022; Khalili et al. 2023). Furthermore, the development of ML surrogate models potentially enables real-time clinical diagnostic tools, transforming complex biomechanical simulations into rapid, actionable insights at the time of care.

### Heterogeneous tissue with multidirectional axons

The brain’s structural complexity, comprised predominantly of neuronal cell bodies in the gray matter and axonal projections forming white matter tracts, contributes to its highly heterogeneous mechanical behavior. While primary injury mechanisms often focus on impact dynamics and head geometries, some secondary injury processes, such as inflammation, edema, and immune activity, are also influenced by the brain’s material heterogeneity. Differences in regional mechanical properties can alter the progression and localization of these injuries, such as the spread of cerebral edema or the recruitment of immune cells.

**Fig. 5:**
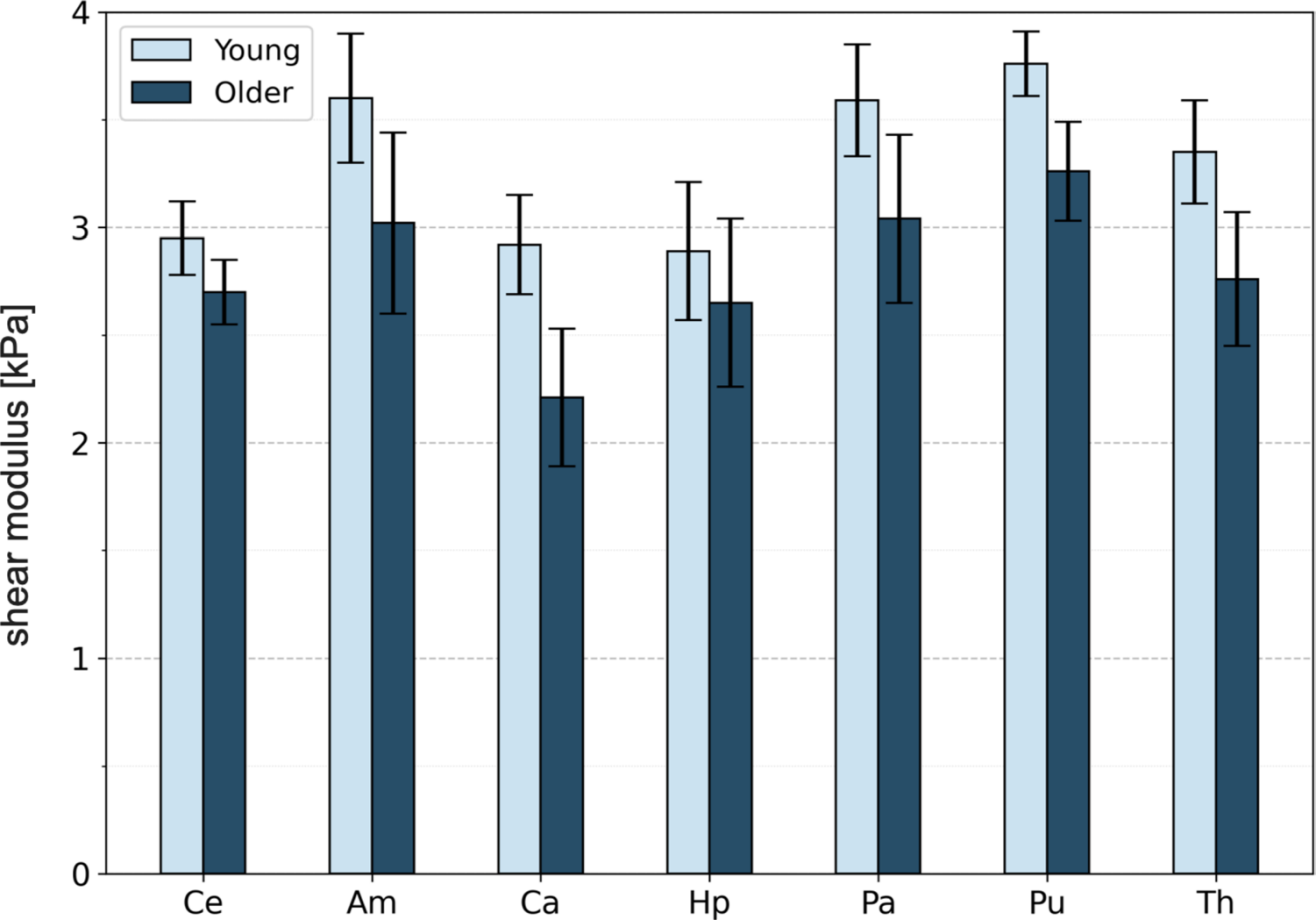
Regional heterogeneity of brain tissue shear moduli for cerebrum (Ce), amygdala (Am), hippocampus (Hp), caudate (Ca), pallidum (Pa), putamen (Pu), and thalamus (Th), in young and older adults (reproduced from Hiscox et al. 2018).

Research has revealed significant variability in material properties across brain regions, with shear stiffness values ranging from 0.6 kPa to 3.0 kPa (Van Dommelen et al. 2010). Later studies showed further variability due to factors like age, region, and sample preparation (Fig. 5) (Budday et al. 2015, 2017; Hiscox et al. 2018; Kwon et al. 2021). The varying tissue-level strains across different brain regions can trigger distinct mechanobiological cascades through secondary injury processes. Areas with higher strain may experience more severe BBB disruption, fluid pressure distribution, axonal injury, and neuroinflammatory responses. For instance, due to their distinct mechanical and structural properties, edema may accumulate differently in gray and white matter. Therefore, while the use of standard adult means remains the established practice due to limited experimental data across different ages and sexes, incorporating a more heterogeneous measure of brain properties could lead to more reliable injury predictions and a more comprehensive understanding of the interplay between cellular and tissue mechanics in TBI.

Mechanical properties can also vary over time throughout the process of injury and recovery; studies suggest that astrocytic responses and the morphological structure result in an increase in the stiffness of the brain tissue immediately after an injury. However, the stiffness decreases in the following 24 hours and returns to the normal level within a week (Feng et al. 2017a; Chen et al. 2020; Eskandari et al. 2021). Finally, the anisotropic nature of white matter also varies significantly between regions depending on the alignment of axonal fibers locally. This anisotropy is particularly relevant to secondary injury because it influences how mechanical strains and stresses propagate, potentially exacerbating damage in specific regions (Sullivan et al. 2015; Sahoo et al. 2016; Labus and Puttlitz 2016; Feng et al. 2017b; Hajiaghamemar et al. 2020; Hajiaghamemar and Margulies 2021).

To better understand secondary injuries due to inflammation, computational models are essential for integrating complex heterogeneous factors that are challenging to capture experimentally. FE models, for example, have been valuable in simulating chronic mechanical forces on brain tissue such as those induced by growing brain tumors. Similarly, we could simulate how regional and directional variations in material properties affect the transmission and distribution of forces (Finan et al. 2017); suggesting that areas with sharp transitions in tissue stiffness (like gray-white matter interfaces) could potentially experience higher stress than their surroundings, leading to local BBB disruption via mechanical deformation of endothelial cells, increased permeability, and disruption of the AQP4 channels. Computational models allow for the incorporation of spatially and temporally varying tissue properties, including both pre-and post-injury conditions, to predict how mechanical forces interact with the microenvironment. Incorporating tissue heterogeneity, such as anisotropic behavior in white matter (Wang et al. 2024; Chavoshnejad et al. 2024; Solhtalab et al. 2025), allows for better predictions of strain distribution, which can be used to predict axonal injury and subsequent neuroinflammation. These models, when combined with experimental data such as imaging techniques, can help in identifying areas of the brain at high risk for secondary injury and in targeting interventions aimed at mitigating damage (Finan et al. 2017; Kwon et al. 2021; Colgan et al. 2010; Chatelin et al. 2011; Giordano and Kleiven 2013; Giordano et al. 2017; Saeidi et al. 2023).

### Complex poroelastic and viscoelastic mechanical behavior

Secondary injuries following TBI often arise from the complex interactions between the fluid and solid components of brain tissue. While brain tissue is typically modeled as a single solid phase in most mechanical simulations (Moran et al. 2014; Wang et al. 2021; Jafarabadi et al. 2023), this simplification overlooks the brain’s true multiphase nature, with approximately 80% fluid and 20% solid medium (Miller 2011). The solid matrix exhibits viscoelastic properties, while the fluid movement within this matrix follows poroelastic behavior (Greiner et al. 2021). The time-dependent behavior of these components plays a crucial role in secondary injury mechanisms such as edema formation, pressure buildup, and the diffusion of inflammatory mediators. Therefore, accurately modeling the nonlinear behavior of brain tissue is essential for predicting intracranial events post-injury and guiding the development of more effective therapeutic strategies.

To address these complexities, a nonlinear multiphasic poro-hyperelastic formulation within the porous media theory framework is required (Biot 1941). This approach accounts for the interaction between the fluid and solid components of brain tissue, providing a more accurate depiction of fluid flow dynamics, mechanical properties, and tissue consolidation under various loading conditions, which is vital for simulating TBI-induced edema formation and subsequent intracranial pressure changes. Studies have used poroelastic models to investigate conditions such as hydrocephalus (Vardakis et al. 2016), ischemia-reperfusion injury (Mokhtarudin and Payne 2017), and even acutely increased ICP immediately after a TBI (Ju et al. 2020). By modeling these fluid-solid interactions, we can better understand how tissue displacement and fluid accumulation influence the progression of various secondary injuries and recovery post-TBI (Wheatley et al. 2015).

In addition, the high stain rates associated with the initial TBI impact, which can reach up to $241 s^{-1}$ during severe injury scenarios (Su et al. 2023), establish a mechanical state that influences the injury progression due to various secondary injury processes. While viscoelasticity is standard in modern computational models to capture this rate-dependent tissue deformation (Miller and Chinzei 2002; Zhang et al. 2024), this integration is often limited to calculating macroscale damage accumulation and immediate biomechanical thresholds. However, the clinical consequences of this deformation extend over longer periods through time-dependent secondary processes. For instance, brain edema can peak over hours to days post-TBI, while neuroinflammation evolves over days to weeks. Similarly, processes like axonal degeneration and glial scar formation unfold over weeks to months. Therefore, the crucial challenge is linking these rate-dependent macroscale inputs to the resulting microscale and multiscale activities. Specifically, there is a need to develop integrated multiphysics frameworks that couple the biomechanical damage, often determined by dynamic FEM simulations, with biological cascades represented by coupled ODEs or reaction-diffusion systems. These frameworks must capture the temporal coupling of initial mechanical changes to the cascading biochemical responses (e.g., calcium influx, sustained microglial activation, and astrocytosis), and fluid dynamics (e.g., dynamic intracranial pressure increases from vasogenic and cytotoxic edema, alongside impaired cerebrospinal fluid clearance) (Vardakis et al. 2016; Su et al. 2023; Anssari-Benam and Saccomandi 2024). This advanced coupling is essential to delineate secondary injury thresholds for diffuse TBI accurately and to predict personalized injury progression beyond immediate impact.

### Repetitive mild TBI

**Fig. 6:**
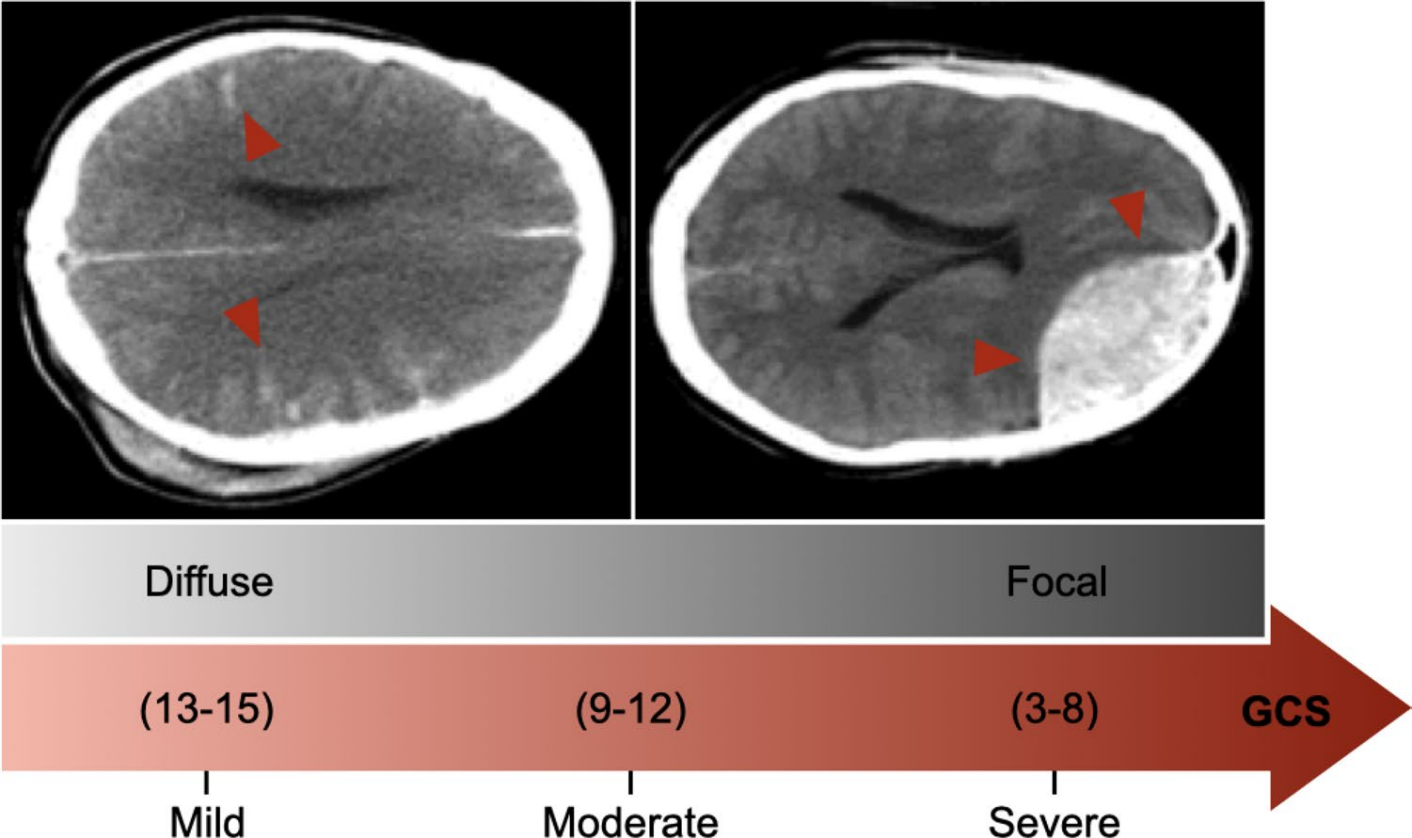
GCS severity classification showing mild, moderate, and severe ranges, with injury patterns categorized as either diffuse or focal brain injury. The scans depicting diffuse (left) and focal (right) injury are adapted with permission from Andriessen et al. 2010.

While early research emphasized moderate to severe TBI, several computational studies have been developed to address mild TBI, particularly in sports, automotive, and military scenarios (Ghajari et al. 2017; Lyu et al. 2022; Ji et al. 2022; Jeffs et al. 2023; Zhang et al. 2004). There is a growing interest in repetitive and sub-injurious head impacts, which remain under-studied both experimentally and computationally (Hajiaghamemar and Margulies 2021; Shi et al. 2021; Boucher et al. 2022). The Glasgow Coma Scale (GCS) rates the severity of a brain injury based on a neurological assessment of a patient’s consciousness afterward; a score of 13–15 is a mild injury, 9–12 is a moderate TBI, and 3 to 8 is a severe TBI (Fig. 6). Most diffuse injuries fall between mild to moderate TBI, while focal injuries are mainly in the severe regime. However, for an injury of a similar impact intensity, a diffuse injury could be more damaging than a focal injury (Andriessen et al. 2010; Keating and Cullen 2021). The general symptoms of mild TBI are usually transient. However, some patients experience long-term neurological impairments, and exposure to additional mild impacts exacerbates the condition.

Repetitive mild TBI increases cellular markers that can initiate the onset or drive the further progression of Alzheimer’s disease (Kimelberg 1995; Weber 2007; Andriessen et al. 2010; Haarbauer-Krupa et al. 2021). Athletes represent the majority of patients who are treated for recurring mild TBI (Bayly et al. 2005; Shahim et al. 2018; Cinelli et al. 2019), although repeated brain injuries in cases of child or domestic abuse are likely underreported.

Mild injuries are typically diagnosed based on acute clinical and behavioral symptoms, as conventional imaging often fails to detect anatomical lesions (Mccrory et al. 2016; Ji et al. 2022; Hoang et al. 2023). Advanced magnetic resonance imaging (MRI) techniques such as diffusion tensor imaging (DTI) have been considered promising tools for the diagnosis of concussion, as the symptoms of a concussion are believed to be associated with changes in the brain microstructure (Eierud et al. 2014). However, assessing changes at the cellular and subcellular levels is difficult, and the results have been inconsistent, showing varying DTI effects depending on cohorts, sports, and concussion history (Vos and Diaz-Arrastia 2015; Afzal and Zaidi 2014).

Although some experimental models study cognitive impairment and axonal injury after repeated mild TBI (Marmarou and Demetriadou 1994; Kane et al. 2012; Shandra and Robel 2020; Shi et al. 2021; Boucher et al. 2022), most computational and experimental models focus on single-impact mild TBI, with fewer addressing the cumulative effects of repetitive or sub-concussive impacts, which are increasingly recognized as critical for long-term outcomes (Shitaka et al. 2011; Shi et al. 2021; Boucher et al. 2022; Chapman et al. 2023). For example, Khalin et al. (2016) used the Glasgow Outcome Scale, commonly used to assess functional neurological/cognitive outcome of TBI patients, alongside a standardized Neurological Severity Score (NSS) to evaluate the cumulative effect of repetitive mild TBIs on cognitive function in rodents, finding detectable cognitive deficits in severe cases one week after injury. Additionally, increasing the time interval between mild TBIs has been shown to protect against long-term conditions, suggesting a vulnerable period during which repeated injuries can lead to lasting effects even at the milder level of injury (Chen et al. 2020; Bodnar et al. 2019). This finding holds significant clinical implications, particularly for athletes at risk for TBI. However, most mild TBI models usually fail to demonstrate high reproducibility rates (Bodnar et al. 2019; Fernández-Liste et al. 2023).

Computational models, including Finite Element, multiphase, and agent-based frameworks, are essential tools for simulating mild TBI and are increasingly being developed to specifically address the cumulative and biological effects of repetitive or sub-injurious impacts (Shi et al. 2021; Miller et al. 2019; Boucher et al. 2022). FE models can simulate the accumulation of mechanical damage by using complex nonlinear constitutive equations of brain tissue deformation under repeated impacts, allowing for systematic variation of injury parameters (impact magnitude, frequency, location) (Miller et al. 2019; Lyu et al. 2022; He and Fan 2023). For instance, these models can simulate a second sub-concussive impact delivered within a short window and predict the elevated axonal strain threshold compared to a single impact. This demonstration of heightened vulnerability in repetitive injuries can be achieved by coupling damage effects with visco-hyperelastic behavior. Validation of such predictions can be achieved by correlating simulated axonal strain with DTI metrics such as fractional anisotropy. These models overcome the ethical and practical limitations of *in vivo* experiments by providing mechanistic explanations for heightened injury vulnerability. Multiphase models, such as poroelastic frameworks, can account for fluid movement and tissue swelling, providing insights into interstitial fluid dynamics and cerebral edema and its contribution to injury progression at microsecond time scales and microscopic spatial scales that are challenging to measure experimentally, especially simultaneously across the whole brain (Vardakis et al. 2016; Basilio et al. 2019; Guo et al. 2020; Corti et al. 2023). Additionally, agent-based models simulate disruptions in cellular signaling, showing how repeated insults can lead to sustained microglial activation and chronic neuroinflammation, which is critical for understanding the persistent biological response to repetitive mild TBI (Beltrán et al. 2023; Wachtler et al. 2025). These predictions can be validated against serum biomarkers (e.g., GFAP, MMP-9, S100B, and UCH-L1) in patients who have sustained repetitive mild TBIs. Importantly, these models should account for a shifted biological baseline, where the cumulative inflammatory response from previous impacts prevents the system from returning to its homeostatic pre-injury state before subsequent impacts occur.

Combining these models in a multiscale framework could link biomechanical thresholds to measurable biomarkers, providing a powerful tool for early diagnosis and guiding therapeutic interventions targeting repetitive injury mechanisms. Finally, with advances in medical imaging and computational power, there is a growing interest in developing patient-specific models to predict injury outcomes and guide personalized treatment plans (Lazaridis et al. 2014; Ghajari et al. 2017). While detailed experimental measurements are often limited to animal models, validated computational models can thus effectively bridge the gap between animal studies and human injury by systematically explaining how injuries accumulate mechanically and biologically over time (Vaughan et al. 2018).

## Conclusions

TBI and its consequences are becoming more widely known; however, accurate assessment of brain status and recovery after injury is more complex. Currently, the diagnosis of traumatic brain injury relies heavily on neurocognitive assessments since the injury might occur predominantly at the cellular level and is not visible in conventional medical imaging modalities. In such cases, computational models could offer an invaluable path forward for injury predictions, but their accuracy and capabilities depend on many factors, including material properties, anatomical and geometric details, boundary conditions, clinical relevance of personalized injury predictions, and, especially their ability to account for mild and repetitive injuries.

To elevate computational modeling into a truly translational tool, future efforts must focus on mechanism-specific advances rather than a single all-encompassing model. Future efforts should prioritize coupling the macro-scale stress and strain predictions to specific outputs, such as linking the localized strain concentration near microvasculature or axonal bundles to the specific critical thresholds for cellular activation that initiate the inflammatory cascade. Similarly, a complementary effort involves models designed to selectively incorporate the most influential aspects of brain heterogeneity, including regional stiffness differences, directional white-matter anisotropy, and time-dependent post-injury remodeling. These mechanism-driven models are necessary to test specific hypotheses about how mechanics shape secondary injury, which is essential for identifying the long-term changes that could lead to neurodegenerative diseases. Additionally, future work should focus on targeted multiphysics approaches that integrate fluid–solid interactions with rate-dependent tissue behavior. These efforts can prioritize specific pathways such as the edema-driven pressure elevations to test how mechanical loading governs longer-term cellular and fluid-dynamic responses. Given the high variability in TBI recovery outcomes, one modeling direction must integrate patient-specific geometry and material properties derived from advanced neuroimaging. The goal is to identify unique anatomical features in an individual patient that increase their susceptibility to cumulative damage from repetitive mild impacts.

The primary value of computational models lies in predicting the underlying biophysical and biological events that contribute to clinical outcomes. Advanced computational frameworks predict mechanistic intermediate variables (e.g., peak axonal strain, cumulative damage, inflammatory mediator concentrations, and BBB disruption) that are often invisible to standard clinical tools. Therefore, the critical step is to rigorously train and validate these mechanistic outputs with actual neurocognitive or long-term neurodegenerative outcomes. Focusing on this fundamental goal enables computational models to become an essential partner, significantly improving the prediction and prevention of future injuries and associated neurodegenerative diseases.

## Data Availability

No datasets were generated or analysed during the current study.
